# Diagnostic Values of Electrocardiogram in Chronic Obstructive Pulmonary Disease (COPD)

**DOI:** 10.4103/0970-2113.44125

**Published:** 2008

**Authors:** R.L. Agarwal, Dinesh Kumar, Gurpreet, D.K. Agarwal, G.S. Chabra

**Affiliations:** Deptt. of Tuberculosis & Chest Diseases, Motilal Nehru Medical College, Allahabad, Uttar Pradesh, India

**Keywords:** Chronic obstructive pulmonary diseases (COPD), Electrocardiographic changes, Sensitivity, Specificity

## Abstract

**Background:**

Chronic obstructive pulmonary diseases (COPD), a broad spectrum of respiratory diseases represents a worldwide problem. Electrocardiographic (ECG) findings may help in clinical decision making regarding this disease entity. Aims: To evaluate the extent and diagnostic values of ECG changes among COPD patients suffering from broad spectrum of respiratory diseases.

**Material & Methods:**

A hospital based cross-sectional study was conducted in Sworoop Rani Nehru Hospital, Allahabad in Eastern Uttar Pradesh (UP), India. A sample of 60 patients attending respiratory diseases OPD for treatment of various respiratory problems including 14 COPD patients was selected randomly during 2000-2001. Patients of respiratory diseases were also evaluated electrocardiographically along with other investigations.

**Results:**

Respiratory problems were more common among rural males of low socio-economic group. COPD particularly chronic bronchitis was the commonest respiratory problem next to pulmonary tuberculosis. Inspite of normal heart rate observed in 71.4% COPD patients, ECG changes were present in 35.7% COPD patients. Peaked P-wave was observed in 35.7% COPD patients, whereas duration of QRS complex was abnormal in only 8.1% of the patients. None of the COPD patients showed abnormal P-wave duration. ECG changes were found less sensitive (35.7%) but highly specific (95.6%).

**Conclusion:**

Diagnostic values of ECG among patients with respiratory problems suggest that COPD patients should be screened electrocardiographically in addition to other clinical investigations.

## INTRODUCTION

In human being, the respiratory and circulatory systems are so intimately related that changes in one sooner or later may cause changes in the other. The various respiratory diseases may secondarily cause changes in the heart, which may be detected by Electrocardiograph (ECG).

Chronic airway obstruction is an important and rapidly increasing problem in different parts of the world. Chronic obstructive pulmonary disease (COPD) is a progressive disease characterized by airflow limitation/obstruction i.e. either not reversible at all or only partially reversible. COPD is associated with abnormal inflammatory response of the lungs to chronic inhalation exposure from smoke, dust and other air pollutants. It manifest as chronic cough with or without sputum production. Chronic bronchitis and emphysema are grouped together as COPD as these conditions cannot be separated^1^–^4^. Manifestation of disease as chronic cough with or without sputum production for more than three months of a year for atleast two consecutive years is considered essential for COPD. It may or may not be accompained with progressive breathlessness. A detailed discussion on guidelines for management of COPD is available in a recent study by Jindal et al^5^.

Varied prevalence of COPD among adult population is reported in India 6–10. Several studies 11–15 reported changes in the activity of heart including P-wave axis and amplitude, rightward displacement of QRS and T-axis, reduction of amplitude of QRS complex in limb and precordial leads, sinus tachycardia, Right bundle branch block (RBBB) etc., among COPD patients. However, COPD patients probably are not usually assessed by electrocardiogram in routine medical practice particularly in developing countries like India. Therefore, the present study was conducted to evaluate the diagnostic values of ECG changes among COPD patients.

## METHODS

Present hospital based cross-sectional study was conducted during July 2000- June 2001 in Swaroop Rani Nehru Hospital, attached with Motilal Nehru Medical College, Allahabad, Uttar Pradesh, India. COPD cases were diagnosed and selected from patients who were attending Outpatient Department of Respiratory Diseases for treatment of various respiratory problems. A total of 60 patients having various respiratory problems were evaluated for COPD.

For diagnosis of COPD, guidelines by American Thoracic Society^1^ and also by British Thoracic Society 2 were followed. COPD included chronic bronchitis and emphysema cases, but asthma was excluded, as airflow obstruction is largely reversible in this condition. Some other chronic lung diseases were also excluded. To assess diagnostic values of ECG, a control group of patients who attended OPD for respiratory problems other than COPD was also considered. Other respiratory diseases like pulmonary tuberculosis, non-tubercular pulmonary infections, pleural disease, malignancy etc were diagnosed by adopting their respective diagnostic criteria available along with clinical judgement.

Information of socio-demographic characteristics like age, sex, religion, socioeconomic status (SES), social background, occupation and smoking habits was collected. Study subjects were classified according to spectrum of respiratory diseases. Patients underwent different clinical and radiological investigations. Further evaluation was also done by ECG. A 12 lead ECG including 3 bipolar limb leads, 3 unipolar limb leads and 6 unipolar precordial leads was performed. All necessary precautions desired in ECG were observed. ECG was done by single channel BPL cardiart various108T/MK-V I machine. Various ECG parameter like rate, axis deviation, P-wave changes, QRS complex, T-wave, ST changes etc. were observed. The axis of P-value and QRS complex was calculated by hexaxial reference system. The study was approved by Institutional research committee and all ethical guidelines of Helsinki^16^ were followed.

## RESULTS

Out of 60 patients with respiratory disease, maximum i.e. 16 (26.7%) belonged to 41-50 years. More than half i.e. 46 (66.7%) were males, with male: female ratio of 2:1. Most of the patients were Hindus i.e. 51 (58.0%), while 09 (15.0%) were Muslim patients. Maximum number of patients i.e. 36 (60.0%) belonged to upper lower socio-economic status, followed by 15 (25.0%) to lower-middle class. More than half i.e. 39 (65.0%) were having rural background, while 21 (35.0%) belonged to urban area. Maximum number of patients i.e. 22 (36.6%) were farmer by occupation, followed by 14 (23.3%) who were housewives. There were 14 (23.3%) cases of COPD among total patients attending the OPD. Mean age of COPD patients was significantly higher than that of patients suffering from other respiratory diseases (Table-1).

**Table 1:** Socio-demographic characteristic of the patient with respiaratory diseases

	Characteristic	No.	%
Age groups (in years)			
	0-10	01	1.7
	11-20	07	11.7
	21-30	11	18.3
	31-40	13	21.7
	41-50	16	26.7
	51-60	07	11.7
	>60	05	8.3
Mean ± SD	37.8 ± 14.24	-	
Sex	Male	40	66.7
	Female	20	33.8
Religion	Hindu	51	85.0
	Muslim	09	15.0
SES	Lower	03	5.0
	Upper Lower	36	60.0
	Lower Middle	15	25.0
	Upper Middle	06	10.0
Background	Urban	21	35.0
	Rural	39	65.0
Occupation	Farmer	22	36.6
	Unskilled laborer	02	3.3
	Skilled laborer	04	6.7
	Business class	03	5.0
	Service class	04	6.7
	Housewives	14	23.3
	Others	11	18.3
	Total	60	100.0
COPD	Present	14	23.3
	Absent	46	76.7
Mean Ages	COPD Present	48.79 ± 10.32	
	COPD Absent	38.25 ± 15.03	P<0.01

Among all the respiratory cases, more than half i.e. 32 (53.3%) were having pulmonary tuberculosis, followed by 14 (23.3%) with COPD and 7 (11.7%) with pleural disease. There were only 2 (3.3%) patient with non-tubercular pulmonary infection. Out of 20(33.3%) smokers, 8 (40.0%) each were patients of pulmonary tuberculosis and COPD respectively (Table-2).

**Table 2:** Distribution of case according to the type of respiratory diseases

Respiratory Diseases	Smokers No. (%)	Total No. (%)
Pulmonary TB	08 (40.0)	32 (53.3)
Non-tubercular Pulmonary Infection	0 (0.0)	02 (3.3)
COPD	08 (40.0)	14(23.3)
Pleural disease	02 (10.0)	07(11.7)
Interstitial Lung Disease	0 (0.0)	02 (3.3)
Tropical pulmonary eosinophilia	01 (5.0)	01 (1.7)
Kyphoscoliosis	0 (0.0)	01 (1.7)
Malignancy	01 (5.0)	01(1.7)

**Total**	**20 (100.0)**	**60 (100)**

Among 14 COPD patients, maximum i.e. 10 (71.4%) patients were having normal heart rate, while 4 (28.6%) were having sinus tachycardia. Out of these 4 patients with sinus tachycardia, 3 (75.0%) were having chronic bronchitis, while 1 (25.0%) was having chronic bronchitis with emphysema. The mean heart rate recorded among COPD patients was 96.14 per minute (Table-3).

**Table 3:** Heart rate in COPD patients

Type of COPD	Heart Rate	
	Normal No. (%)	Sinus Tachycardia No. (%)

Chronic Bronchitis + Emphysema	04 (40.0)	01 (25.0)
Emphysema	03 (30.0)	0 (0.0)
Chronic Bronchitis	03 (30.0)	03 (75.0)

**Total**	**10 (100.0)**	**04 (100.0)**

As far as the ECG changes in COPD patients are concerned, ECG changes were present in half i.e. 5 (35.7%) COPD patients, while 2 (4.4%) ECG changes were recorded among patients not having COPD. The duration of P-wave was normal in all cases of COPD. The peaked P-wave was present in 5 (35.7%) of COPD cases, while in only 2 (4.4%) of the cases in which COPD was absent, peaked P-wave was observed. More than half i.e. 9 (64.3%) COPD patients had normal P-wave axis. Only 5 (35.7%) patients with COPD had left-axis deviation (LAD) or right axis deviation (RAD) or indeterminate P-wave axis, while majority of patients i.e. 45 (97.8%) in whom COPD was absent had normal P-wave axis. The duration of QRS complex was normal in 13 (92.9%) patients with COPD. All the COPD patients had normal QRS amplitude Majority i e 12 (85 7%) COPD patients had normal QRS axis, while LAD/RAD/Axis in North-west zone was recorded in only 2 (14.3%) patients. Most of the patients i.e. 13 (92.9%) with COPD reported normal ST-T segment. Sensitivity and Specificity of ECG findings were found to be 35.7% and 95.6% respectively (Table-4).

**Table 4:** ECG changes in COPD patients

ECG Changes	COPD Present	COPD Absent
**P-wave**		
Duration		
Normal	14 (100.0)	46 (100.0)
Abnormal	0 (0.0)	0 (0.0)
Amplitude		
Normal	09 (64.3)	44 (95.6)
Peaked	05 (35.7)	02 (4.4)
Axis		
Normal	09 (64.3)	45 (97.8)
LAD/RAD/ Indeterminate	05 (35.7)	01 (2.2)
**QRS Complex**		
Duration		
Normal	13 (92.9)	46 (100.0)
Abnormal	01 (8.1)	0 (0.0)
Amplitude		
Normal	14 (100.0)	45 (97.8)
Low	0 (0.0)	01 (2.2)
Axis		
Normal	12 (85.7)	45 (97.8)
LAD/RAD/ Axis in NWZ	02(14.3)	01 (2.2)
**ST-T Changes**		
Normal	13 (92.9)	44 (95.6)
Inverted T-wave/Depression of ST segment	01 (8.1)	02 (4.4)
**ECG Changes**		
Present	05 (35.7)	02 (4.4)
Absent	09 (64.3)	44 (95.6)

**Total**	**14 (100.0)**	**46 (100.0)**

## DISCUSSION

Among 60 patients with respiratory disease enrolled in the present study, maximum number of patients belonged to 41-50 years. The prevalence was higher among males, Hindus, belonging to upper lower socio-economic status, living in rural areas and who were farmers. Out of 14 cases of COPD, 40% were smokers. The mean heart rate among COPD patients was 96.14 per minute. Sinus tachycardia was recorded in about one-fourth of the COPD patients. Fifty percent of the COPD patients had ECG changes. More than one-third of the COPD patients had peaked P-waves and abnormal P-waves axis. Only one patient was recorded as having abnormal duration of P-wave. Inverted T-wave was also observed in only one patient. None of the patient recorded abnormal duration of P-wave and abnormal amplitude of QRS complex.

The mean heart rate in the present study was recorded as 96.14 per minute as compared to 86 per minute obtained by Calatayud et al^11^. Normal sinus rhythm was recorded in 57.1% cases. Sinus tachycardia was present in 28.6% cases. Scott RC et al^12^ reported arrhythmias other than sinus tachycardia to be uncommon in chronic corpulmonale.

In the present study, peaked P-wave i.e. amplitude more than 2.5 mm, was recorded in 35.7% of the cases with COPD. In Spodicks^13^ series, 13.9% of COPD patients had P-wave equal or greater than 2.5mm. Carid and Wilcken^14^ found incidence of P-pulmonale in 15.5% of their COPD patients, while Scott et al^15^ and Pinto et al^17^ recorded same incidence of 32.7% in their studies. In the present series, in COPD patients, 35.7% deviation in the axis was recorded. Among all, 21.4% patients had a mean P-wave axis value of +60° or more. Caird et al^14^ and Chappell et al^18^ reported an axis of +70° or more in 79% and 29% cases respectively. Only 7.1% case had slight (+30°) LAD of P-wave axis.

None of the cases in the present series showed low QRS amplitude in frontal and left precordial leads. The QRS axis was within normal range in 85.7% cases as compared to only in 27.8% found by Phillips et al^19^. Slight left axis deviation (−30°, and −15°, respectively) of mean QRS axis was present in 14.3% cases. Calatayud et al^11^ showed prevalence of LAD of QRS in 12.1% cases. T-wave inversion in leads V_1_ to V_3_ was found in only 7.1% cases. The case had associated RVH and incomplete RBBB and the inversion could be attributed to them. Pinto et al^17^ found T-wave inversion in 18.5% cases.

The ECG findings were found to be 35.7% sensitive and 95.6% specific in diagnosis of COPD among patients having respiratory problems. So, there are chances of false negative but not of false positives in detecting COPD cases by ECG. Based on the findings of the study, Positive predictive value was found to be 71.4% meaning thereby that the chances of COPD among patients having ECG changes are high. Similarly, negative predictive value was 83.0%, meaning thereby that the chances of not having COPD among patients not having ECG changes are also quite high. Bayesian approach for predicting probability of COPD among cases having abnormal ECG findings can also be attempted for updating clinical decisions in view of prior clinical experiences regarding patients with respiratory problems. In clinical practice, cases having respiratory problems especially COPD should also be assessed for ECG changes and our decision should be supplemented by those findings.
